# Estimating overannotation across prokaryotic genomes using BLAST+, UBLAST, LAST and BLAT

**DOI:** 10.1186/1756-0500-7-651

**Published:** 2014-09-16

**Authors:** Gabriel Moreno-Hagelsieb, Brigitte Hudy-Yuffa

**Affiliations:** Department of Biology, Wilfrid Laurier University, 75 University Ave. W, N2L 3C5 Waterloo, ON Canada

**Keywords:** Annotation quality, Overannotation, Pairwise alignment, Sequence comparison, NCBI’s BLAST, BLAST+, UBLAST, LAST, BLAT

## Abstract

**Background:**

As the number of genomes in public databases increases, it becomes more important to be able to quickly choose the best annotated genomes for further analyses in comparative genomics and evolution. A proxy to annotation quality is the estimation of overannotation by comparing annotated coding genes against the SwissProt database. NCBI’s BLAST (BLAST+) is the common software of choice to compare these sequences. Newer programs that run in a fraction of the time as BLAST+ might miss matches that BLAST+ would find. However, the results might still be useful to calculate overannotation. We thus decided to compare the overannotation estimates yielded using three such programs, UBLAST, LAST and the Blast-Like Alignment Tool (BLAT), and to test non-redundant versions of the SwissProt database to reduce the number of comparisons necessary.

**Findings:**

We found that all, UBLAST, LAST and BLAT, tend to produce similar overannotation estimates to those obtained with BLAST+. As would be expected, results varied the most from those obtained with BLAST+ in genomes with fewer proteins matching sequences in the SwissProt database. UBLAST was the fastest running algorithm, and showed the smallest variation from the results obtained using BLAST+. Reduced SwissProt databases did not seem to affect the results much, but the reduction in time was modest compared to that obtained from UBLAST, LAST, or BLAT.

**Conclusions:**

Despite faster programs miss sequence matches otherwise found by NCBI’s BLAST, the overannotation estimates are very similar and thus these programs can be used with confidence for this task.

**Electronic supplementary material:**

The online version of this article (doi:10.1186/1756-0500-7-651) contains supplementary material, which is available to authorized users.

## Findings

### Background

The continuing growth of the number of genome sequences in public databases has become an always present meme in the introduction to most bioinformatics articles. It is therefore important to develop fast methods for analyzing such amounts of genomic data. Overannotation, an estimate on the proportion of false genes annotated into a genome, can work as a proxy to genome annotation quality (see examples of use at [1–6]). In this regard, Skovgaard *et al.* [7] developed a method to estimate the number of genes that should be annotated in a genome. The method is based on comparing the proteins encoded by the annotated genes of a genome against the SwissProt database [8, 9] (see Data and methods for further details).

In a mini review about the 1000th genome deposited into public databases, Lagesen *et al.* [10] made a point about the time required to analyze a high number of genomes. In the case of estimating overannotation by the SwissProt method, the bottleneck would be in comparing the annotated coding genes to the SwissProt database, which is commonly performed, like many other protein sequence comparisons, using some version of NCBI’s BLAST [11, 12]. However, there has been a few new programs promising to do a much faster work comparing sequences, such as the BLAST-Like Alignment Tool (BLAT) [13], LAST [14], and the sequence analysis multitool USEARCH [15], which contains UBLAST as a substitute for BLAST. While these algorithms produce results faster, the speed comes at the cost of missing some proportion of similar sequences. However, the calculation of overannotation by the SwissProt method depends on the proportion of large and small annotated proteins that find a match in the SwissProt database, rather than on the total number of sequences finding matches, and rather than on the number of matches found by each sequence. Therefore, even if these new sequence comparison tools miss matches that would be found by BLAST, the relative proportions of small and large proteins finding matches in the SwissProt database might be similar and, thus, render these newer sequence comparison tools just as useful and more efficient for the task of estimating overannotation.

In this work, we tested the performance of BLAT, LAST and UBLAST, for the specific task of estimating overannotation as compared against NCBI’s BLASTP+ [12]. Since SwissProt contains redundant sequences, we also tested if we could reduce the database, by eliminating nearly identical sequences, without losing information towards estimating overannotation.

### Data and methods

The version of the SwissProt database [8, 9] available by early December 2013 contained a total of 540,261 protein sequences. The quality of these sequences is annotated in a five level hierarchy. We wrote a program to remove any protein sequence with qualities 4 (Predicted) and 5 (Uncertain) leaving 522,651 sequences. The same program reduced the database by keeping only one example of any identical protein sequences taking the above 522,651 to 438,166 non-identical sequences, thus reducing the number of sequences in the database by approx. 16%. The UCLUST function of USEARCH7.0.959 (32 bit-compiled, which is the free version for academic and non-profit institutions) was used to produce SwissProt databases with only one representative for very similar sequences by clustering at 95, 90, 85, 80, 75 and 70% identity thresholds.

We estimated the overannotation of approx. 2700 prokaryotic genomes available at the RefSeq database [16] (ftp://ftp.ncbi.nih.gov/genomes/Bacteria/) by early December 2013.

The version of NCBI’s BLAST (BLAST+) was 2.2.28+ (64 bit-compiled), LAST version was 392 (64 bit-compiled), BLAT was version 32 (64 bit-compiled). The version of UBLAST was the so named function implemented under USEARCH7.0.959 (kindly provided by the author 32 bit-precompiled). Both BLAST+ and UBLAST were run with default parameters, except for an E-value threshold of 1e-6. BLAT was run with default parameters. The first experiments, those used to compare processing speeds, were run in a late 2012 iMac. This computer was not running any other process during these experiments. Calculations for all 2700 genomes using BLAST+ were run in computer clusters kindly provided by SHARCNET. All the genome-to-SwissProt comparisons using the faster programs were run at the late 2012 iMac.

Overannotation was calculated using the SwissProt method described by [7]. Briefly, the method estimates the number of genes that should be annotated in a genome by calculating the proportion of genes coding for proteins at least 200 amino-acid residues long (deemed as true genes), matching proteins in the SwissProt database (large SP-matching genes); and the proportion of small annotated genes, those that would code for proteins less than 200 amino-acid residues long, also matching SwissProt proteins (small SP-matching genes). The proportions are expected to be very similar if there is no overannotation. The lower the proportion of small SP-matching genes compared to that of large SP-matching genes, the higher the overannotation.

### Results

As mentioned, filtering out protein sequences labelled either “Predicted” or “Uncertain” from the SwissProt database left 522,651 sequences, while eliminating identical sequences left 438,166 sequences. Further clustering sequences using USEARCH’s UCLUST function left 339,818 sequences at 95%, 299,959 at 90%, 268,285 at 85%, 239,682 at 80% 214,044 at 75% and 190,445 at 70% identity thresholds.

**Figure 1:**
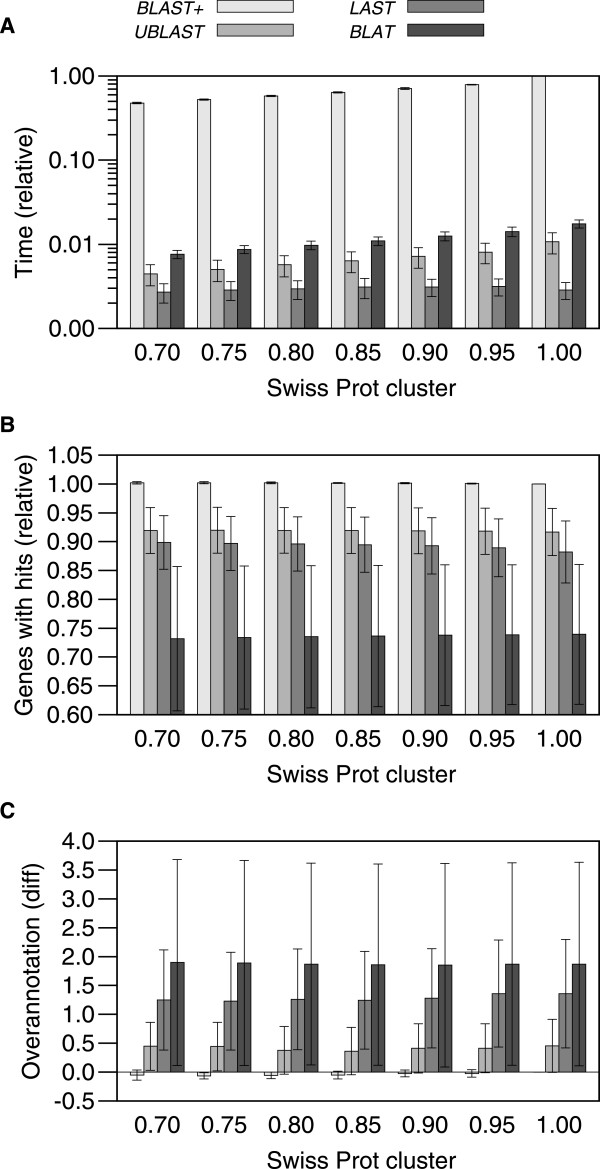
**Ten-genomes experiment.** **(A)** UBLAST, LAST and BLAT ran in less than a hundredth of the time as NCBI’s BLAST, with LAST running the fastest; **(B)** All these programs matched fewer genome proteins to proteins in the SwissProt database than BLAST+, with BLAT showing the lowest numbers and the highest variation; **(C)** UBLAST produced the most similar overannotation estimates to those produced by BLAST+, while BLAT produced the most dissimilar ones. Filtering the SwissProt database at different identity thresholds did not have much of an effect in speed or in overannotation estimates.

To get an initial view of the possibility to using UBLAST, LAST and/or BLAT to estimate overannotation we chose ten initial test genomes from the RefSeq genome dataset (Table 1). We made sure to have genomes of different lengths and different overannotation estimates (as previously determined with BLAST+). We ran BLAST+, UBLAST, LAST and BLAT on those genomes against all the SwissProt databases with reduced redundancies mentioned above, giving us a combination of three programs times seven SwissProt databases = 21 experiments per genome. Time saving was calculated from the output of the UNIX *time* command (our results are based on *Real* time, *System* and *User* times can be found in Additional file 1).The initial experiments showed that all faster algorithms, UBLAST, LAST and BLAT, run in less than a hundredth of the time as BLAST+ (Figure 1A). All algorithms produced fewer proteins matching sequences in the SwissProt database (Figure 1B), with BLAT producing the lowest numbers. Overannotation estimates were similar to those obtained with NCBI’s blast, except for a tendency for BLAT to produce around 10% higher estimates (Figure 1C). BLAT produced the results varying the most from those obtained with BLAST+.Reduced SwissProt databases did not change the results obtained too much (Figure 1). The variation from results using a non-identical SwissProt database increased slightly with the threshold for producing non-redundant databases. However, in terms of time to run, reducing the SwissProt databases did not have as much of an appreciable effect as using the faster algorithms.

**Table 1:** **Genomes used in the ten-genomes experiment**

Organism (NCBI’s UID)	SP hits	Genes	Gene estimate	Overannotation (%)
*Streptococcus pneumoniae* 670-6B (52533)	1430	2352	1750	34.40
*Bacillus cereus* ATCC 10987 (57673)	3399	5844	4305	35.75
*Bacillus subtilis* 168 (57675)	3170	4176	3568	17.04
*Escherichia coli* K-12 MG1655 (57779)	3670	4145	3936	5.31
*Burkholderia pseudomallei* 1710b (58391)	3818	6344	5578	13.73
*Burkholderia cenocepacia* MC0-3 (58769)	4829	7008	5971	17.37
*Anaeromyxobacter* sp. K (58953)	2623	4457	3837	16.16
*Methylobacterium nodulans* ORS2060 (59023)	4107	8308	5819	42.77
*Coprothermobacter proteolyticus* DSM5265 (59253)	1016	1482	1239	19.61
*Lactobacillus salivarius* CECT5713 (162005)	1176	1552	1485	4.51
*Mycobacterium abscessus* GO06 (170732)	2443	2626	2580	1.78

Gene estimates and overannotation as calculated with BLAST+ [7].

Given the results of the experiments above, we further tested the difference in overannotation estimates for all the remaining prokaryotic genomes available in our database with all four programs (BLAST+, UBLAST, LAST and BLAT). We did not calculate time differences because BLAST+ would take too long to run in our machines. BLAST+ experiments were run in computer clusters. Since the time saved using reduced SwissProt databases was minimal, we made all of these comparisons against the clean SwissProt database with 438,166 non-identical sequences.

**Figure 2:**
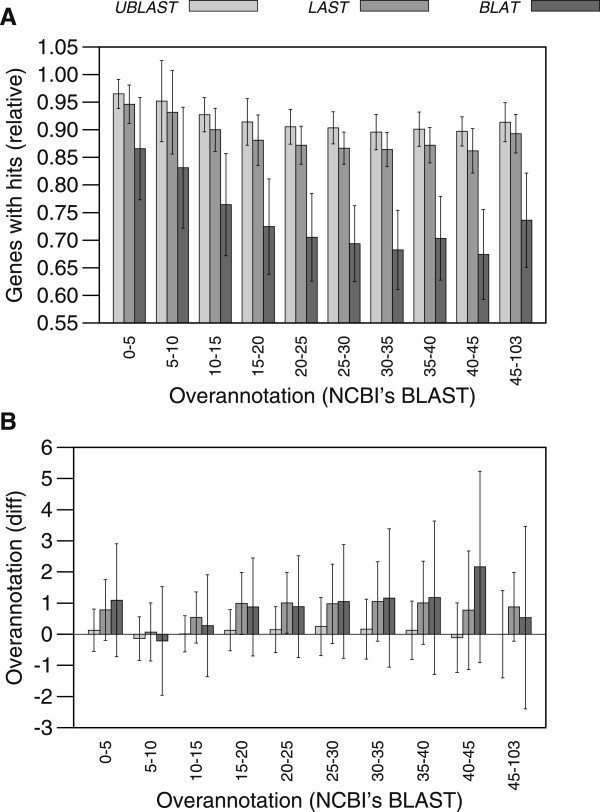
**Effect of overannotation.** **(A)** As in the small experiment, the faster programs tended to match a lower proportion of genome proteins to the SwissProt database than BLAST+, with BLAT missing the highest proportion of matches. **(B)** The difference between the overannotation estimates produced by the faster programs compared to those produced by BLAST+ tended to be small and increase only modestly with the original overannotation estimate.

Results with all the available prokaryotic genomes confirmed that all three faster programs, UBLAST, LAST and BLAT, produce a lower number of proteins with matches in the SwissProt database. With UBLAST getting the closest results to those obtained with BLAST+ (Figure 2A). The difference in the overannotation estimates tended to increase slightly with the original overnnotations as calculated with NCBI’s BLAST+ (Figure 2B). However, the difference remained small and within a range of less than five (complete sets of overannotation estimates are included in Additional files 2, 3, 4 and 5).

**Figure 3:**
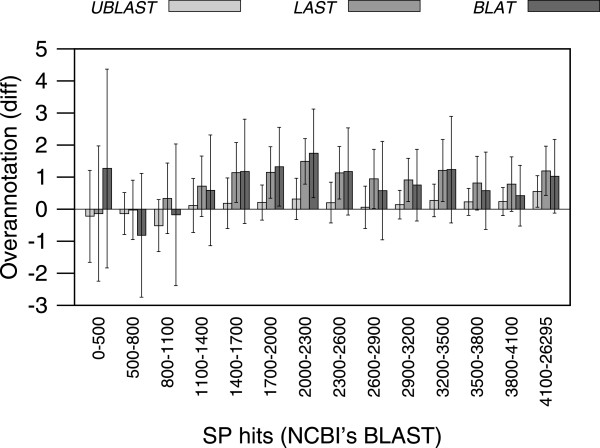
**Effect of matches to SwissProt.** The difference in overannotation estimates, as compared against those produced with BLAST+, seems more pronounced for genomes with fewer proteins finding matches in the SwissProt database. This effect is more noticeable with BLAT.

Overannotation estimate differences against those obtained with BLAST+ seemed to concentrate at genomes with fewer BLAST+ matches in the SwissProt database (Figure 3). This result is to be expected, since a smaller sample should increase the probability of making mistakes, and the effect of such mistakes would be more evident. However, results with genomes containing at least 800 encoded proteins with matches in the SwissProt database resulted in overannotation estimates showing little variation compared to BLAST+. Overall, results using UBLAST were less variable from BLAST+ results than those obtained with BLAT, with LAST results in the middle (Figures 2B, and 3) (Paired t-tests for overannotation estimates: UBLAST *vs.* BLAST+: *t* = 7.12 *p* < 1.5∗10^-12^; LAST *vs.* BLAST+: *t* = 40.93, *p* < 2.2∗10^-16^; BLAT *vs.* BLAST+: *t* = 19.62, *p* < 2.2∗10^-16^).

## Electronic supplementary material

Additional file 1: **Run times.** Run times for each experiment. Ten genomes, four programs. Times included are real, user and system times as reported by the time built-in command of the bash shell. (ZIP 4 KB)

Additional file 2: **Overannotation estimates (BLAST+).** Tab-separated tables with total annotated gene counts, total gene estimates as calculated from the proportion of genes whose proteins match proteins in the SwissProt database, and the resulting overannotation as a percent of the total gene estimate. (ZIP 31 KB)

Additional file 3: **Overannotation estimates (BLAT).** Tab-separated tables with total annotated gene counts, total gene estimates as calculated from the proportion of genes whose proteins match proteins in the SwissProt database, and the resulting overannotation as a percent of the total gene estimate. (ZIP 31 KB)

Additional file 4: **Overannotation estimates (LAST).** Tab-separated tables with total annotated gene counts, total gene estimates as calculated from the proportion of genes whose proteins match proteins in the SwissProt database, and the resulting overannotation as a percent of the total gene estimate. (ZIP 31 KB)

Additional file 5: **Overannotation estimates (UBLAST).** Tab-separated tables with total annotated gene counts, total gene estimates as calculated from the proportion of genes whose proteins match proteins in the SwissProt database, and the resulting overannotation as a percent of the total gene estimate. (ZIP 31 KB)
